# The importance of pre-training gap analyses and the identification of competencies and skill requirements of medical personnel for mass casualty incidents and disaster training

**DOI:** 10.1186/s12889-021-10165-5

**Published:** 2021-01-09

**Authors:** Krzysztof Goniewicz, Mariusz Goniewicz, Anna Włoszczak-Szubzda, Frederick M. Burkle, Attila J. Hertelendy, Ahmed Al-Wathinani, Michael Sean Molloy, Amir Khorram-Manesh

**Affiliations:** 1 Department of Aviation Security, Military University of Aviation, 08-521 Dęblin, Poland; 2 Department of Emergency Medicine, Medical University of Lublin, 20-059 Lublin, Poland; 3 University of Economics and Innovation, Lublin, Poland; 4 Harvard Humanitarian Initiative, T.H. Chan School of Public Health, Harvard University, Boston, MA 02115 USA; 5 Department of Information Systems and Business Analytics, College of Business, Florida International University, Miami, FL 33174 USA; 6 Department of Emergency Medical Services, Prince Sultan Bin Abdulaziz College for Emergency Medical Services, King Saud University, Riyadh, 11451 Saudi Arabia; 7 Department of Emergency Medicine, Wexford General Hospital, Wexford, Y35 Y17D, Ireland; 8 School of Medicine, University College Dublin, Donnybrook, D4 Ireland; 9 BIDMC Fellowship in Disaster Medicine, Department of Emergency Medicine, Beth Israel Deaconess Medical Centre, 457 Brookline Avenue, Boston, MA 02215 USA; 10 Institute of Clinical Sciences, Department of Surgery, Sahlgrenska Academy, Gothenburg University, 413 45 Gothenburg, Sweden; 11 Research Advisor, Department of Development and Research, Armed Forces Center for Defense Medicine, 426 76 Gothenburg, Västra Frölunda Sweden

**Keywords:** Hospital preparedness, Hospitals, Disaster training, Mass casualty incidents, medical personnel

## Abstract

**Background:**

Effective preparedness to respond to mass casualty incidents and disasters requires a well-planned and integrated effort by all involved professionals, particularly those who are working in healthcare, who are equipped with unique knowledge and skills for emergencies. This study aims to investigate and evaluate the level of knowledge and skills related to mass casualty and disaster management in a cohort of healthcare professionals.

**Methods:**

A cross-sectional brief study was conducted using a validated and anonymous questionnaire, with a sample of 134 employees at a clinical hospital in Lublin, Poland.

**Results:**

The findings of this study may indicate a need for standardization of training for hospitals employees. It also suggests a knowledge gap between different professional groups, which calls for adjusting such general training, to at least, the weakest group, while special tasks and mission can be given to other groups within the training occasion.

**Conclusion:**

Pre-Training gap analyses and identification of participants’ competencies and skills should be conducted prior to training in mass casualty incidents and disasters. Such analyses provides an opportunity to develop training curriculum at various skill and knowledge levels from basic to advance. All training in mass casualty incidents and disasters should be subject to ongoing, not just periodic, evaluation, in order to assess continued competency.

**Supplementary Information:**

The online version contains supplementary material available at 10.1186/s12889-021-10165-5.

## Background

The medical outcomes of Mass Casualty Incidents (MCI) depend on the resilience of health care systems, defined as the 4Rs, i.e. Robustness (infrastructure and human resilience), Redundancy (the availability of material resources and the competences of health care personnel), Resourcefulness (the existence of plans and strategies), and Rapidity (the prompt setting of priorities) [1–4]. Hospital preparedness constitutes both structural and non-structural readiness. Structural readiness encompasses facilities, buildings and other infrastructural assets. Non-structural readiness deals with strategies and plans.

In addition to structural and non-structural preparedness, effective hospital preparedness requires an effectively trained and experienced healthcare workforce to manage crisis incidents. Numerous studies have identified deficiencies in knowledge and experience among hospital staff. Therefore, robust training standards need to be developed for healthcare workers in order to successfully manage future MCI’s or disaster [5–9].

Operational knowledge is normally acquired through a course of didactic study. This knowledge converts to skills through drilling and practical exercises. Desired competences comprise the knowledge and skills acquired as a result of effectively training healthcare personnel to become qualified professionals capable of understanding mass casualty incidents and disasters in their entirety, thereby acting responsibly and effectively in crises.

Many researchers, dealing with the effectiveness of training related to mass casualty incidents and disasters, place emphasis on the organisational competence of individual employees as well as on individual teamwork skills. However, healthcare professionals should also demonstrate leadership, conflict avoidance, and management skills [10–15]. These skills and competencies should be evaluated continuously to improve both individual and team performance. Therefore, competency evaluation is a vital component of both initial and refresher training to ensure competencies are achieved and maintained. Such competency evaluations thus contribute to successful performance during mass casualty incidents and disasters. It verifies the effectiveness of completed training, the retention of knowledge and skills of healthcare workers, and can be used to determine perceptions of self-competence, reinforcing learning. Numerous studies suggested that health care workers, confident in their own high level of competence, are more likely to react effectively in real crisis situations and more often than workers who perceive their competence as being low [16–22].

The aim of this study was to gain insights on the training needs of health care workers with regard to preparedness (knowledge and skills) for mass casualty incidents and disasters.

## Methods

### Location of the study

A quantitative cross sectional brief study was conducted from February 10–12, 2020 at Public University Hospital No. 1 in Lublin, Poland. This hospital was designated as one of the Coronavirus Disease 2019 (COVID-19) hospitals and therefore was selected for this cross-sectional brief study. It has 550 beds (including chemotherapy beds). It employs approximately 1600 people (including physicians, nurses, allied health personnel, and administrative, technical, cleaning and service personnel) [23]. The most recent published statistical data for the academic year 2017/2018 revealed the number of admissions to be approximately 19,000 patients, the number of surgical procedures performed was approximately 19,000 [23]. The average length of stay (LOS) was 4.4 days. The hospital includes an infectious diseases ward, where COVID-19 patients from the Lublin region are currently being admitted and treated [23].

### Study population

A quantitative cross-sectional brief study was conducted in the form of an anonymous and voluntary survey sent to 134 health care workers, constituting 11.1% of the total number of health care workers employed at Public University Hospital No. 1 [23]. All respondents agreed to participate in the survey. More than half of the surveyed were women (56%; *N* = 75). Men constituted 44% (*N* = 59) of the study group. Healthcare workers who had over 20 years of work experience (41.8%; *N* = 56) comprised the largest group of survey respondents, followed by workers with experience of 6–10 years (19.4%; *N* = 26), 0–5 years (16.4%; *N* = 22), 11–15 years (12.7%; *N* = 17) or 16–20 years (9.7%; *N* = 13) [23]. Physicians (41.8%; *N* = 56) and nurses (46.3%; *N* = 62) were the largest groups in this study, followed by 11.9% (*N* = 16) of paramedics.

### Survey design

The initial questionnaire was developed based on a literature review by the authors. The following keywords: disaster hospital preparedness; disaster training; disaster medical personnel were searched using PubMed, Scopus and Web of Science search engines. Resulting data was then organized, categorized, and mapped. A qualitative methodology was used to verify the research tool. We piloted our questionnaire (Supplementary file 1) on 10 healthcare professionals to determine if the questions were appropriately constructed for readability, and study feasibility. Pilot data was subsequently excluded from the final study analysis. The original questionnaire was developed for this study and was designed to take no more than 15 min to complete [23]. Both open-ended and close-ended questions were utilized to capture and generate relevant data and to provide an opportunity for respondents to answer questions in their own words. The participants received information about the study. The information included the study’s purpose, the voluntary nature of their participation, and strict confidentiality and secure data storage. It complied with the ethical principles stipulated by Polish law and thus was exempted from ethics approval requirements.

### Study format

Due to the prevailing COVID-19 pandemic restrictions the authors’ questionnaire was available both in hard copy and online and included 18 closed questions with two further open-ended questions. Respondents were asked about their own competency experiences, prior courses and training, as well as knowledge about the preparedness of the workplace for mass casualty incidents and disasters. There were 2 questions, which aimed to assess the perceived preparedness quantitively (questions 12 and 13). Each question in this group was formulated as a statement, which could be answered using a Likert scale from 1 to 10, where 10 meant Very well and 1 meant Very low.

### Data analysis

Due to current Coronavirus 2019 infection, this study could only be performed in one hospital. Therefore, no power calculation was performed. The remaining statistical analyses were conducted using IBM SPSS Statistics version 26. Frequency analyses, analysis of basic descriptive statistics, correlation analysis with Spearman’s Rho coefficient, chi square independence tests, Student’s t-test for dependent samples, one-factor analysis of variance, and Student’s t-tests for independent samples along with Mann Whitney tests were conducted. Statistical significance level was set at α = 0.05 [23].

## Results

### Relationship between sociodemographic variables

Sociodemographic variables and the relationship between the evaluation of preparedness for disasters and mass casualty incidents and disasters were assessed [23]. Length of service was positively correlated with the evaluation of preparedness of the current workplace for mass casualty incidents and disasters (weak relationship). The longer the respondents have been working, the better the preparedness of the facility where they work for mass casualty incidents and disasters. There was no statistical significance in terms of gender. The professional background (physician, nurse, and paramedic) had no statistically significant difference in the evaluation of one’s own preparedness (Table 1).

**Table 1:** Evaluation of individual preparedness, and that of the workplace, for a mass casualty incident or disaster, by profession

	doctor (*n* = 56)		nurse (*n* = 62)		paramedic (*n* = 16)		*F*	*p*	ω^2^
	*M*	*SD*	*M*	*SD*	*M*	*SD*			
Evaluation of the preparedness of the current workplace for a mass-casualty incident or disaster	3.82	1.40	4.49	1.83	3.56	1.71	3.37	0.037	0.03
Evaluation of individuals personal preparedness for a mass-casualty incident or disaster	6.07	2.01	5.39	2.32	5.94	1.95	1.56	0.213	0.01

*M* Mean, *SD* Standard deviation; *p* significance; *ω2* estimation of variance

More than half of the respondents (53.7%; *N* = 72) had not received training related to preparation for mass casualty incidents and disasters in their current workplace. Only 46.3% (*N* = 62) of respondents declared to have received such training.

### Procedures for dealing with mass casualty incidents and disasters in the workplace, and respondents’ knowledge of same

A series of chi square tests of independence were performed in order to check the relationship between the knowledge of the procedures for dealing with mass casualty incidents and disasters, and the knowledge of persons responsible for directing operations, procedures and logistical resources in the workplace.

### Knowledge of persons responsible for directing operations during mass casualty incidents and disasters in the workplace

A statistically significant effect was observed between the knowledge of the person responsible for directing operations during mass casualty incidents and disasters in the workplace, and the knowledge of procedures for dealing with mass casualty incidents and disasters: χ2(1) = 56.39; *p* < 0.001; Vc = 0.65.

A larger percentage of respondents who were familiar with the operating procedures were able to identify those responsible for directing operations in the event of a mass casualty incident or disaster. The effect size is large (Table 2).

**Table 2:** Knowledge of persons responsible for directing operations during mass casualty incidents and disasters in the workplace, and knowledge of the subject

Knowledge of the person responsible for directing operations during mass casualty incidents and disasters in the workplace	Have you been familiarised with the procedures to be followed in the event of a mass casualty incident or disaster?			
	Yes		No	
	*N*	%	*N*	%
Yes	97	93.3%	9	30.0%
No	7	6.7%	21	70.0%

The relationship between knowing what to do in the event of a mass casualty incident at one’s workplace and knowing the procedures to be followed in the event of a mass casualty incident or disaster was then examined. A statistically significant effect of large size was obtained: χ2(1) = 32.78; *p* < 0.001; Vc = 0.50.

People who are familiar with the procedures for dealing with mass casualty incidents and disasters more frequently declared knowing how to proceed in such situations in their workplace (Table 3).

**Table 3:** Knowledge of the rules of conduct in situations of mass casualty incidents and disasters at your workplace, and knowledge of the operating procedures in this area

Knowledge of the rules of conduct in the event of evacuation during a mass casualty incident and disaster at your workplace	Have you been familiarised with the procedures to be followed in the event of a mass casualty incident or disaster?			
	Yes		No	
	*N*	%	*N*	%
Yes	100	96.2%	17	56.7%
No	4	3.8%	13	43.3%

There was a significant correlation between having adequate logistical resources to deal with mass casualty incidents at the workplace, and knowledge of the operating procedures in the event of a mass casualty incident or disaster. The effect size was moderate: χ2(2) = 17.82; *p* < 0.001; Vc = 0.37. Comparisons were made of the column proportions with Bonferroni correction. A larger percentage of people unfamiliar with the procedures for dealing with these situations do not know whether their workplace is logistically prepared for a mass casualty incident or disaster. In addition, a greater proportion of those who were familiar with procedures for dealing with mass casualty incidents and disasters felt that their workplace had the appropriate logistical resources to deal with such situations (Table 4).

**Table 4:** Having adequate logistical resources in the event of a mass casualty incident at the respondent’s workplace, and knowledge of the procedures to be followed in this respect

Does your workplace have adequate logistics resources for mass casualty incidents?	Have you been familiarised with the procedures to be followed in the event of a mass casualty incident or disaster?			
	Yes		No	
	*N*	%	*N*	%
Yes	50	48.1%	3	10.0%
No	20	19.2%	5	16.7%
Don’t know	34	32.7%	22	73.3%

#### Knowledge of operating procedures in the event of a mass casualty incident or disasters and evaluation of one’s own preparedness, and that of the workplace, in this regard

A series of tests were performed to check for differences in the evaluation of preparedness for a mass casualty incident or disaster, depending on whether the test subjects were familiar with the rules of conduct in such situations. Due to the large numerical differences between the subgroups, the analysis was based on Mann Whitney tests.

We tested to see whether having an action plan for mass casualty incidents and disasters in the facility where the subjects worked differentiates the evaluation of preparedness for mass casualty incidents and disasters (Table 5).

**Table 5:** Evaluation of individuals personal preparedness, and that of the workplace, for a mass casualty incident or disaster, and the existence of an action plan for the institution

	Does the facility where you currently work have an action plan for dealing with mass casualty incidents and disasters?				*Z*	*p*	η^2^
	Yes (*n* = 96)		Don’t know (*n* = 34)				
	average rank	*Me*	average rank	*Me*			
Evaluation of preparedness of the current workplace for a mass casualty incident or disaster	70.21	4.00	50.44	3.50	2.69	0.007	0.06
Evaluation of individuals personal preparedness for mass casualty incidents and disasters	71.20	6.00	49.41	5.00	2.93	0.003	0.07

Mann Whitney test results were statistically significant for both variables. Those who reported that the facility they worked in had an action plan for dealing with mass casualty incidents and disasters, evaluated both their own preparedness, and that of the facility, for a mass casualty incident or disaster better than those who did not know whether their facility had such a plan. The effect size was moderate. Analogous tests were conducted to examine the differences between persons who were familiar with the operating procedures in the event of a mass casualty incident or disaster, and persons who were not familiar with these procedures in terms of their evaluation of preparedness for a mass casualty incident or disaster (Table 6).

**Table 6:** Evaluation of individuals personal preparedness, and that of the workplace, for a mass casualty incident or disaster, and familiarisation of the respondent with the operating procedures in this respect

	Have you been familiarised with the procedures to be followed in the event of a mass casualty incident or disaster?				*Z*	*p*	η^2^
	Yes (*n* = 104)		No (*n* = 30)				
	average rank	*Me*	average rank	*Me*			
Evaluation of preparedness of the current workplace for a mass casualty incident or disaster	74.29	4.00	41.97	3.00	−4.11	< 0.001	0.13
Evaluation of individuals personal preparedness for mass casualty incidents and disasters	70.72	6.00	56.35	5.00	−1.81	0.071	0.02

The tests performed showed a statistically significant effect for the evaluation of preparedness of the current workplace for a mass casualty incident or disaster. This means that people who have been acquainted with the procedure for dealing with mass casualty incidents and disasters do a better job of evaluating the preparedness of their current workplace for such situations than respondents who have not been acquainted with the procedures. The size of this effect was moderate.

Statistically significant effects were also noted, both for the evaluation of the preparedness of the current workplace and for one’s own preparedness. The respondents who were able to identify the person responsible for directing operations in such situations did a better job of evaluating their own preparedness and that of the facility than the respondents who did not know who was responsible for directing operations during mass casualty incidents and disasters. The size of these effects was moderate (Table 7).

**Table 7:** Evaluation of individuals personal preparedness, and that of the workplace, for mass casualty incidents and disasters, and knowledge of the person responsible for directing operations

	Do you know who is responsible for directing operations for mass casualty incidents and disasters in the facility where you work?				*Z*	*p*	η^2^
	Yes (*n* = 106)		No (*n* = 28)				
	average rank	*Me*	average rank	*Me*			
Evaluation of preparedness of the current workplace for a mass casualty incident or disaster	72.60	4.00	46.00	3.00	3.30	0.001	0.08
Evaluation of individuals personal preparedness for mass casualty incidents and disasters	72.39	6.00	48.98	5.00	2.87	0.004	0.06

We also examined whether knowledge of the rules of conduct in the event of an evacuation during a mass casualty incident at their workplace differentiates between evaluations of preparedness for a mass casualty incident or disaster. Mann Whitney test results showed statistically significant differences between the compared groups. Those who knew the rules of conduct in the event of an evacuation during a mass casualty incident at their workplace were better at evaluating both their own preparedness and that of the facility for mass casualty incidents and disasters than those who did not know these rules. The size of the effect for the evaluation of preparedness of the current workplace was moderate, while for one’s own preparedness it was small (Table 8).

**Table 8:** Evaluation of individuals personal preparedness, and that of the workplace, for mass casualty incidents and disasters, and respondents’ knowledge of the rules of conduct in this respect

	Do you know the rules of conduct in the event of evacuation during a mass casualty incident at your workplace?				*Z*	*p*	η^2^
	Yes (*n* = 117)		No (*n* = 17)				
	average rank	*Me*	average rank	*Me*			
Evaluation of preparedness of the current workplace for a mass casualty incident or disaster	70.78	4.00	41.24	3.00	−3.00	0.003	0.07
Evaluation of individuals personal preparedness for mass casualty incidents and disasters	70.29	6.00	48.32	5.00	−2.20	0.028	0.04

#### Having adequate logistical resources for mass casualty incidents and disasters, and the evaluation of individuals personal preparedness, and that of the workplace, in this regard

One-factor analysis of variance was performed to determine whether the answers to the question of whether the facility where the subjects worked possessed adequate logistical resources to deal with mass casualty incidents impacts upon the evaluation of preparedness for such situations (Table 9).

**Table 9:** Having adequate logistical resources for mass casualty incidents and disasters, and the evaluation of individuals personal preparedness, and that of the workplace

	Does your workplace have adequate logistical resources for mass casualty incidents?						*F*	*p*	ω^2^
	Yes (*n* = 53)		No (*n* = 25)	Don’t know (*n* = 56)					
	*M*	*SD*	*M*	*SD*	*M*	*SD*			
Evaluation of preparedness of the current workplace for a mass casualty incident or disaster	4.75	1.60	3.52	1.42	3.75	1.69	7.24	0.001	0.09
Evaluation of individuals personal preparedness for mass casualty incidents and disasters	6.28	1.86	5.96	2.46	5.13	2.17	4.27	0.016	0.05

The results of this analysis showed statistically significant effects for both variables. In order to examine the exact differences, Sidak post hoc tests were performed. The respondents who claimed that their workplace had adequate logistical resources for a mass casualty incident or disaster were better able to evaluate their own preparedness, and that of the facility, when compared to the respondents who did not know whether their facility had adequate logistical resources (Table 10).

**Table 10:** Significance levels of post hoc tests for evaluations of preparedness for mass casualty incidents and disasters, depending on whether the facility had adequate logistical resources

	Evaluation of preparedness of the current workplace for a mass casualty incident or disaster		Evaluation of individuals personal preparedness for mass casualty incidents and disasters	
	Yes	No	Yes	No
No	0.006	0	0.896	0
Don’t know	0.005	0.910	0.015	0.277

### Plans for dealing with mass casualty incidents and disasters at the current workplace, and availability of training in this regard

The relationship between having an action plan to deal with mass casualty incidents and disasters in the workplace, as well as the availability of training, was examined. A statistically significant effect of small size was observed: χ2(1) = 10.12; *p* = 0.001; Vc = 0.28.

The respondents who declared that no exercises had been organised concerning procedures to be followed during mass casualty incidents and disasters in the facility, more often than not, did not know whether the facility had a plan for dealing with such situations (Table 11).

**Table 11:** Organisation of exercises for mass casualty incidents and disasters, and having an action plan at the current workplace

Have there been any disaster preparedness exercises organised at the facility where you currently work?	Does the facility where you currently work have a plan for dealing with mass casualty incidents and disasters?			
	Yes		Don’t know	
	*N*	%	*N*	%
Yes	53	55.2%	8	23.5%
No	43	44.8%	26	76.5%

We examined whether the frequency of training was linked to whether the current workplace had a plan for dealing with mass casualty incidents and disasters. There was no statistically significant correlation. In the last stage of the analysis, we tested to see whether training related to preparation for a mass casualty incident or 1disaster in the current workplace was linked to whether the workplace had a plan for dealing with mass casualty incidents and disasters. A statistically significant effect of small size was observed: χ2(1) = 5.19; *p* = 0.023; Vc = 0.20.

A larger percentage of people who had not received training did not know whether their facility had an action plan for these situations (Table 12).

**Table 12:** Having a plan of action for mass casualty incidents and disasters by the current workplace, and the relationship to training

Have you received training in preparation for a mass casualty incident or disaster (including epidemics) at your current workplace?	Does the facility where you currently work have a plan for dealing with mass casualty incidents and disasters?			
	Yes		Don’t know	
	*N*	%	*N*	%
Yes	50	52.1%	10	29.4%
No	46	47.9%	24	70.6%

### Evaluation of respondents’ training needs for mass casualty incidents and disasters

The questions concerning opinions on the need to organise training, as well as the requirements for training by the employing facility, the variable proved to be almost constant, thus, these issues were not included in the statistical analysis. Instead, they are important from the point of view of qualitative research, in relation to the training needs of the respondents. The majority of respondents (99.25%; *N* = 133) believe that the employer should organise training related to responding to mass casualty incidents and disasters, and that the employer should require employees to undergo such training (96.26%; *N* = 129).

## Discussion

The main outcomes of this study may indicate a gap in knowledge, skills and competency between various groups of healthcare professionals who are tasked to participate in the management of mass casualty incidents and disasters. This gap should be identified and addressed through implementing a comprehensive training and education strategy to strengthen healthcare providers’ ability to effectively respond to an emergency.

The pathway to acquiring disaster management competencies is to obtain appropriate knowledge, and skills that are reinforced through consistent drills and evaluations [24]. Earlier studies have shown that healthcare workers with higher educational levels, gain more confidence through training thus reacting more precisely and responsively to emergencies [23]. Consequently, the main goal in any healthcare emergency preparedness educational initiative, for a group with varied professional backgrounds and knowledge, should be to increase the entire groups’ collective and individual knowledge demonstrably.

Many disaster-training courses aim to deliver a didactic level of knowledge, which only consider the educational backgrounds of participants and not their knowledge in disaster management. In reality, various professionals, such as those in this study, may participate in educational initiatives without having any knowledge of disaster management principles, i.e., command, control, communication, coordination and collaboration. The result would be theoretical knowledgeable professionals, who cannot collaborate or communicate. Therefore, this study suggests that a pre-training evaluation should be performed before any educational initiative to identify any gaps that may exist between different participating professional groups [25–27]. Such an evaluation provides an opportunity to scale the training to begin with the most basic level of knowledge and skills and progressing to more advanced levels of information [28].

Prior to any training for mass casualty incidents and disasters, it is necessary to define the desired competencies. Without them, it is difficult to determine the content and methodology of the proposed training. It is also difficult to examine the needs of respondents in this respect. Well formulated competencies constitute the basis for building effective and targeted training. The teaching and training of competencies is based on integrated medical education, and the number of competency profiles for health care professionals may vary depending on the profession being trained [28].

This study, in accordance to earlier studies, may highlight the need to provide standardized training for hospital employees in order to manage mass casualty incidents and disasters effectively [29–32]. In this study, although all participants declared their perception of preparedness in their current workplace, they had obviously more than one workplaces. Working at several hospitals necessitate a standardization of training which match to all hospitals and provide adequate response of well-trained staff irrespective of their workplace. Previous scholars have discussed the importance of standardization when creating teams capable of working together in a crisis. Best practices in disaster management response suggest that healthcare professionals should acquire knowledge and skills in: collaboration (being an effective team member, coping with crisis situations); professionalism (professional and ethical); communication (building communication channels, building trust in/from patients/team, controlling information flow) [33–37]. Specifically, the basic competencies in training concerning mass casualty incidents and disasters include, but are not limited to, recognising potential critical events, implementing actions, understanding institutional plans for crisis situations and demonstrating skills and knowledge required to perform particular tasks during a disaster [38, 39].

This study has also shown that the longer the respondents’ length of service the better they evaluated their own preparedness, and that of the hospital, for mass casualty incidents and disasters. Several earlier studies have focused mainly on the importance of knowledge and skill growth after training and have not attached importance to the experience gained from length of service [40–42]. Experience should thus be taken into consideration when planning training initiatives.

In addition, the profession of respondents clearly influenced the evaluation of preparedness for mass casualty incidents and disasters. Nurses and paramedics rated the preparedness of their current workplace for mass casualty incidents and disasters, the highest and the lowest, respectively. The evaluation of one’s own preparedness was not statistically significant, but it was the physicians and paramedics, respectively, who evaluated this item as the highest. Other studies have shown that nurses, most often, had the lowest level of knowledge and skills, and trust in leaders [43–48].

Respondents who declared a high self-assessment of knowledge and skills for mass casualty incidents and disasters, in most cases, better evaluated the preparedness of the current workplace. Previous research on the relationship between self-assessment of trainees and their actual knowledge and skills, disclosed that to a large extent, it is the attitudes and beliefs of hospital staff which impact upon their readiness to work in conditions associated with mass casualty incidents and disasters [6, 10, 49–52].

Finally, this study assessed the educational needs of hospital medical staff and found that training methods, their frequency and the examination of the state of knowledge of the trainees, immediately after the training and over time, are important for the effectiveness and sustainability of educational efforts. Existing literature on this subject state that the best results are achieved through repetition of training (at least once a year). This, so-called “tailor-made training” (training prepared on the basis of research into the current needs of the recipients of such training) also uses Blended Learning strategies in the form of theory, skills and attitude training supplemented by multifaceted simulation. Such training should also be evaluated in all its phases after the completion of the training and over time [10, 47, 53–59].

The presented analysis has also some limitations. The main limitation of this study is its single institutional nature, since employees of only one hospital were tested. The COVID-19 pandemic proved to be an obstacle to engage more hospitals and employees in this research and forced the survey to be conducted both on paper and online. Despite these limitations, the survey identified gaps and training needs in the preparation of medical personnel for mass casualty incidents and disasters. The authors’ experience gained from this study will form the basis for planned future studies with further hospitals, and a more comprehensive approach. At the same time, it serves a wider standardisation of the research tool used.

Another limitation of the study was the small number of paramedics surveyed (11.9%), which made the direct comparisons across the three groups more problematic. The small number of paramedics in this study was because they mainly work in the field and the possibilities of reaching them are limited.

A further limitation to this study is the self-assessment questionnaire. Although it can be an inexpensive, practical, fast, scalable, comparable, easy to analyse, standardized way of obtaining confidential data, it can also be associated with dishonest, incomplete answers with interpretation and analysis issues, lack of personalization, response inconsistency, and survey fatigue. Finally, the small sample size (134 people) limits the ability to draw systemwide conclusions applicable for multiple hospital or regional comparisons. However, having accumulated valuable experience from responders, the survey gains mandate to be used for examination of a larger and diverse group of health care professionals and enables the authors to form the basis for planned future research. During the nationalization of the survey, its content will be discussed and modified, if necessary, to serve as a wider standardisation of the research tool.

## Conclusions

There is a gap in knowledge, skills and competencies between various groups of staff in a hospital with respect to mass casualty incident and disaster management.

Training for mass casualty incidents and disasters should be conducted regularly and refreshed at intervals. In order to improve hospital preparedness and resiliency, disaster management competencies should be linked to the overall hospital quality improvement process, with particular emphasis on the members of the health care staff with the shortest length of service. Although an inter-agency approach characterizes the chains of disaster management, the in-hospital training should be tailored towards specific medical professions (nurses, doctors, paramedics), and not for health care workers in general. In order to ensure the appropriate level of knowledge and skills of hospital medical personnel for mass casualty incidents and disasters, it is necessary (as a minimum) to design training curriculum that is comprehensive and aligns with international standards that include the roles, responsibilities, functions and resources needed for MCI and disaster preparedness and response. The provision of training for mass casualty incidents and disasters by the employer should be mandatory, as should the participation of employees (verified by the employer).

## Supplementary Information

**Additional file 1.** Questionnaire. Original developed questionnaire for this study (English version).

## Data Availability

The datasets used and/or analysed during the current study are available from the corresponding author on reasonable request.

## Abbreviations

MCI: Mass Casualty Incidents
COVID-19: Coronavirus Disease 2019
LOS: Length of stay
