# Supplementary material for: The importance of pre-training gap analyses and the identification of competencies and skill requirements of medical personnel for mass casualty incidents and disaster training
Source: BMC Public Health. 2021 Jan 9;21:114. doi: 10.1186/s12889-021-10165-5 (PMC7796807; doi:10.1186/s12889-021-10165-5)
Supplement: Supplementary file 1 — Additional file 1. Questionnaire. Original developed questionnaire for this study (English version). [file 12889_2021_10165_MOESM1_ESM.pdf]

Appendix 1

Questionnaire

Please read the questionnaire carefully and fill it in. When answering, please tick the appropriate boxes. The survey is completely anonymous. Your responses will only be used for statistical summaries.

1. **Gender:**
   - ☐ Men
   - ☐ Women
2. **Length of service:**
   - ☐ Up to 5 years
   - ☐ 6-10
   - ☐ 11-15
   - ☐ 16-20
   - ☐ 20 years and over
3. **Occupation:**
   - ☐ psyhician
   - ☐ nurse
   - ☐ paramedic
4. **Does the facility where you currently work have an action plan for dealing with mass casualty incidents and disasters?**
   - ☐ Yes
   - ☐ No
   - ☐ I don't know
5. **Have you been familiarised with the procedures to be followed in the event of a mass casualty incident or disaster?**
   - ☐ Yes
   - ☐ No
6. **Do you know who is responsible for directing operations for mass casualty incidents and disasters in the facility where you work?**
   - ☐ Yes
   - ☐ No
7. **Do you know the rules of conduct in the event of evacuation during a mass casualty incident at your workplace?**
   - ☐ Yes
   - ☐ No

8. **Does your workplace have adequate logistics resources for mass casualty incidents**?

- ☐ Yes
- ☐ No
- ☐ I don't know

9. **Have there been any disaster preparedness exercises organised at the facility where you currently work?**

- ☐ Yes
- ☐ No

10. **Have you received training in preparation for a mass casualty incident or disaster (including epidemics) at your current workplace?**

- ☐ Yes
- ☐ No

11. **Do you have knowledge of triage?**

- ☐ Yes
- ☐ No

12. **Evaluation of the workplace, for a mass casualty incident or disaster in scale 1 to 10**

Very low 1 2 3 4 5 6 7 8 9 10 verry well

13. **Evaluation of individual preparedness, for a mass casualty incident or disaster, in scale 1 to 10**

14. Very low 1 2 3 4 5 6 7 8 9 10 verry well

Thank you for completing the survey
